# Teachers’ and students’ perceptions on barriers and facilitators for eHealth education in the curriculum of functional exercise and physical therapy: a focus groups study

**DOI:** 10.1186/s12909-019-1778-5

**Published:** 2019-09-06

**Authors:** M. M. Wentink, P. C. Siemonsma, L. van Bodegom-Vos, A. J. de Kloet, J. Verhoef, T. P. M. Vliet Vlieland, J. J. L. Meesters

**Affiliations:** 1 Department of Orthopaedics, Rehabilitation and Physical Therapy, Leiden University Medical Centre, Leiden, The Netherlands; 2 Faculty of Health, Amsterdam University of Applied Sciences, Amsterdam, The Netherlands; 3 Faculty of Health Care, University of Applied Sciences , Leiden, The Netherlands; 4 Department of Biomedical Data Sciences, section Medical Decision Making, Leiden University Medical Centre, Leiden, The Netherlands; 5 Faculty of Health, Nutrition and Sports, The Hague University of Applied Sciences, The Hague, The Netherlands; 6 Innovation, Quality + Research, Basalt Rehabilitation Centre, The Hague / Leiden, The Netherlands

**Keywords:** eHealth, Education, Students, Learning, Technology, Curricula

## Abstract

**Background:**

Despite the growing importance of eHealth it is not consistently embedded in the curricula of functional exercise and physical therapy education. Insight in barriers and facilitators for embedding eHealth in education is required for the development of tailored strategies to implement eHealth in curricula. This study aims to identify barriers/facilitators perceived by teachers and students of functional exercise/physical therapy for uptake of eHealth in education.

**Methods:**

A qualitative study including six focus groups (two with teachers/four with students) was conducted to identify barriers/facilitators. Focus groups were audiotaped and transcribed in full. Reported barriers and facilitators were identified, grouped and classified using a generally accepted framework for implementation including the following categories: innovation, individual teacher/student, social context, organizational context and political and economic factors.

**Results:**

Teachers (*n* = 11) and students (*n* = 24) of functional exercise/physical therapy faculties of two universities of applied sciences in the Netherlands participated in the focus groups. A total of 109 barriers/facilitators were identified during the focus groups. Most related to the Innovation category (*n* = 26), followed by the individual teacher (*n* = 22) and the organization (*n* = 20). Teachers and students identified similar barriers/facilitators for uptake of eHealth in curricula: e.g. unclear concept of eHealth, lack of quality and evidence for eHealth, (lack of) capabilities of students/teachers on how to use eHealth, negative/positive attitude of students/teachers towards eHealth.

**Conclusion:**

The successful uptake of eHealth in the curriculum of functional exercise/physical therapists needs a systematic multi-facetted approach considering the barriers and facilitators for uptake identified from the perspective of teachers and students. A relatively large amount of the identified barriers and facilitators were overlapping between teachers and students. Starting points for developing effective implementation strategies can potentially be found in those overlapping barriers and facilitators.

**Registration:**

The study protocol was a non-medical research and no registration was required. Participants gave written informed consent.

**Electronic supplementary material:**

The online version of this article (10.1186/s12909-019-1778-5) contains supplementary material, which is available to authorized users.

## Background

Application of eHealth, which is defined as ‘the use of new Information and communication technologies (ICT) to improve or support health and health care’ [1], varies largely in health care, e.g. web and mobile applications, electronic patient records, health-sensors and wearable devices, telecommunication, home automation and robotics and serious gaming [1]. Physical therapists mainly use eHealth to support patients in maintaining independency in daily functioning but also for health care processes and services (e.g. telemedicine and electronic patient files) [2].

The availability of technology in health care is growing and there is an urgent need for health professionals who can use eHealth competently and confidently in clinical practice. This means that there is large responsibility for the institutions for the education of future health professionals to ensure that students acquire knowledge, skills and attributes to work with eHealth, and this requires revision of curricula of education [3, 4]. Students should be actively taught how to find, understand, apply and appraise eHealth innovations [5] to constantly update their skills and knowledge [2, 6, 7]. Ideally, students should become early adaptors and lead eHealth initiatives in settings where eHealth adoption is still low [8].

Despite the growing importance of eHealth in the work field, curricula are currently underdeveloped in teaching eHealth in the field of e.g. dietetics, nursing, occupational therapy, physiotherapy, psychology, or social work [5–7, 9, 10]. Therefore, a systematic approach to design, teach, assess or accredit eHealth education in the curriculum is needed. In the literature, a number of barriers for the uptake of eHealth education in curricula were identified: outdated and rigid curricula with narrow focus on technology [7], teachers’ limited experience with and knowledge of the emerging field of eHealth [2, 10, 11] and health care teachers not feeling confident with technology [2, 12, 13].

There are several gaps in the knowledge of uptake of eHealth education in the curriculum. First, research about eHealth education predominantly comes from the medical and nursing literature [8]. However, the paramedical education for physical and functional exercise therapy, should also equip students to confidently use of eHealth, especially because eHealth innovations are increasingly used in daily practices of the physical and exercise therapist (e.g. Fysiogaming, eExercise, activity tracking, etc.). Since the work and patient groups of physical therapists differ from those of nurses, it is expected that barriers and facilitators for eHealth education are different from those already identified in studies in medical and nursing literature. Second, there is a need for more in-depth knowledge of barriers and facilitators for the uptake of eHealth in education, in terms of the factors that may critically influence uptake [14]. Third, previous studies mainly focused on single groups of teachers, students or professionals although it would be interesting to simultaneously contrast teachers’ and students’ points of view on barriers and facilitators for eHealth education.

This study aims to provide insight in the barriers and facilitators for uptake of eHealth in the education for physical therapy and functional exercise therapy, more specific to answer the research question: what are the barriers and facilitators perceived by teachers and students for implementing eHealth in education?

## Methods

### Design

A qualitative study was conducted among teachers and students to explore the perceived barriers and facilitators for uptake of eHealth education in the curricula of the education for physical therapy and functional exercise therapy. ‘EHealth education’ was defined as teaching how to provide treatments using technology. Focus groups were conducted to collect data that contributes to a better understanding of teachers’ and students’ attitudes, experiences with and expectations of eHealth in education [Kitzinger, 2006]. Participants were informed that data would remain confidential and would be anonymously used for scientific research and for improvement of eHealth education. The COnsolidated criteria for REporting Qualitative research (COREQ) guidelines were used for adequate reporting of the study [15].

### Recruitment and inclusion

Teachers and students were recruited from two departments teaching functional exercise therapy and physical therapy in a 4 years full-time program, in the Netherlands: (1) Functional Exercise Therapy, Faculty of Health, University of Applied Sciences in Amsterdam (HvA) and (2) Physical Therapy, Division of Health Care, University of Applied Science in Leiden (HSL). Functional Exercise Therapy and Physical Therapy have similarities (both focus on restoring activities of daily life by means of exercises), but also have differences and are seen as two different paramedical health professions in the Netherlands. Two different health care educations from two different universities of applied sciences were included to ensure a diversity in the population of this study to improve the transferability of the findings.

Teachers from the departments of functional exercise therapy or physical therapy were included if they were: 1) working as a health professional, OR 2) working as a researcher in the field of eHealth. Students were able to participate if they were in year 3 or 4 of the study and completed successfully their placement. Thus both teachers and students were able to reflect on the eHealth education in the curriculum as well as on requirements for successful use of eHealth in clinical practice. Teachers and students were invited to participate via the internal web page and a short oral presentation. Those who were willing to participate, received an email with study information, an informed consent form, and were invited for the focus group.

### Focus groups

The focus groups took place in October and November 2016 at the Universities of Applied Sciences in Amsterdam and in Leiden. Separate groups were organized for teachers and students to ensure that both groups could talk freely about their experiences with eHealth in education. Group size was 5–8 participants to include a diversity of opinions and perspectives, and to allow optimal interaction between participants [16]. The focus groups were conducted by 1) a moderator (MW, female), 2) an assistant (student 1, female) who supported the moderator and managed the tape-recorders and time, and 3) an observer (student 2, female) who took notes and made sure every participant was given the opportunity to speak freely. The moderator has a master’s degree in Health Sciences and functional exercise therapy and had formal training in conducting focus groups. The moderator was a colleague of some of the participating teachers and a former teacher of some of the participating students. Participants did not receive reimbursement for their participation.

An interview guide was developed based on the implementation model of Grol and Wensing. This model was chosen, since it offers a framework to identify and categorize barriers and facilitators for the uptake of innovations within a specific context, in this case the uptake of eHealth education in the curriculum. The framework includes six levels: Innovation (e.g. advantages, feasibility, accessibility, attractiveness of eHealth), Individual (e.g. motivation, awareness, knowledge, skills and attitude of students and teachers), Social context (e.g. opinion of colleagues, work culture), Organizational context (e.g. organization of the curriculum, capacities, resources, structures) and Political and Economic factors (e.g. financial arrangements, regulations, policies). By including questions according to each level of the framework the research team aimed to contribute to the need for more in-depth knowledge of factors (barriers and facilitators) that may critically influence the uptake of eHealth in education [8].

At the beginning of each focus group a brief description of eHealth was given. Open-ended questions within each level were asked to facilitate interactions and in depth discussion between the participants about eHealth education [17]. Examples of questions are: “What do you need in order to be able to use eHealth in education?” or “Why would you use eHealth in your lessons?”. Prompts were used (e.g. pictures expressing emotions) to facilitate participants in verbalizing thoughts. The interview guide was discussed and pilot tested in a group of students. The focus groups were planned to last approximately 1 hour and were aimed to continue until data saturation was reached (not more than two new subthemes retrieved from the focus groups).

### Ethical issues and approval

All participants gave written informed consent prior to participation. Participants were informed that their statements were confidential, would be used for research and to improve eHealth education and would not affect their position as a teacher or student.

### Data analysis

Focus groups were audiotaped, transcribed in full and analyzed using direct content analysis. The implementation framework of Grol and Wensing (2004) is often used for implementing interventions and innovations in health care. Because the framework is highly structured and generally accepted in the field of implementation it was used in this study to structure and describe barriers and facilitators for implementing eHealth in education fromthe perspective of both students and teachers [18]. First step in the analyses was to identify barriers and facilitators for each level of the framework (innovation, individual teacher, individual student, social context, organizational context and political and economic factors) by initial coding of quotes. Second, quotes with comparable content for barriers and facilitators were categorized into (sub)themes. These (sub)themes were further analyzed and categorized into main themes. Data analysis was performed by two students who independently coded and categorized the data. Each step of the data analyses was discussed among the students until consensus was reached. The completed analyses was verified by a third researcher [MW]. Again, discrepancies were discussed among students and researcher until consensus was reached. Microsoft Office Excel was used for data analysis.

## Results

### Participants

Eleven teachers and 26 students indicated their willingness to participate and were invited for the focus groups. Two students were not present, since they had forgotten the appointment. A total of six focus groups was conducted, two with teachers (*n* = 11) and four with students (*n* = 24). Table 1 presents participants’ characteristics.

**Table 1:** Characteristics of the participants in the focus groups

Characteristics	Teachers (*n* = 11)	Students (*n* = 24)
Age, median (range)	38 (29–52)	23 (20–25)
Gender; male, yes (%)	5 (45)	15 (63)
Year in study, number (%)		
Third year	–	13 (54)
Fourth year	–	11 (46)
Profession, number (%)*		
Exercise therapy	3 (27)	–
Physical therapy	7 (64)	–
Health Sciences / Human Movement Sciences	4 (36)	–
Working experience in years, number (%)		
2–3	2 (18)	–
3–4	4 (36)	–
>4	5 (46)	–
Working as a health professional, yes (%)	7 (64)	–
Working as a researcher, yes (%)	4 (36)	–

*Multiple answers possible

### Framework

In the first step of the analyses, a total number of 109 barriers and facilitators (codes) were retrieved from the six focus groups with teachers and students, from which 44 were overlapping between teachers and students, 27 were only identified by teachers and 38 only by students. Next, the barriers/facilitators were organized into 51 subthemes (see Additional file 1) and 14 main themes within the six levels of the framework of Grol and Wensing (see Table 2).

**Table 2:** Results on codes, themes and levels of perceived barriers (B) and facilitators (F) for eHealth education according to teachers and students

			Teachers		Students	
Codes (*n* = 109)	Themes	Level	B	F	B	F
26	Unclear concept of eHealth	Innovation	X		X	
	Lack of a quality mark and evidence for eHealth services.		X		X	
17	Capabilities of students on how to use eHealth	Individual student	X	X	X	X
	Attitude/behavior of students towards eHealth			X	X	X
22	Capabilities of teachers on how to use eHealth	Individual teacher	X	X	X	
	Attitude/behavior of teachers towards eHealth (education)		X	X	X	
14	Inefficient use of expertise	Social context	X		X	
	Communities of practice			X		
	Interprofessional collaboration/education		X	X	X	X
20	(Lack of) a shared vision within the organization	Organizational context	X	X		
	Situational factors (e.g. lack of time, slow curricula changes)		X	X		
10	Financial aspects (e.g. no reimbursement, time and money investment)	Economic and political context	X		X	
	Role of the government (e.g. quality mark for eHealth, reimbursement)			X		X
	Role of profession bodies (e.g. provision of education for therapists)					X

In the following sections, the main themes within each level of the framework will be discussed, first those themes identified by both teachers and students, then those for teachers only and then those for students only.

### The innovation

*Unclear concept of eHealth* was mentioned by both teachers and students. Both groups report it is important for students’ to learn to motivate what, when and how to use eHealth in the treatment process of a patient, and to learn to match available eHealth with the preferences of a patient and to provide support to patients with using eHealth. Both students and teachers referred to eHealth by mentioning applications, electronic patient records and digital exercises. Other available eHealth applications (e.g. virtual reality, robotics/ house automation) were barely mentioned. When discussing eHealth tools, both teachers and students wondered whether they were aware of all the possibilities.

*Lack of a quality mark and evidence for eHealth services* was reported by both teachers and students. Both groups related the lack of a quality mark (i.e. applicability, usability, content, privacy, safety) and the relative absence of evidence for eHealth interventions to a lack of eHealth in education. *“Teacher K: It is tricky. You can never implement something new in education if you waiting for the scientific evidence. The paradox with eHealth is that you are not going to know until you give it a try”.*

### The individual student

*Capabilities to use eHealth* was rated highly by teachers, since they agreed that current generations of students are in general competent with using technology. However, this does not imply that students are also able to innovate health care through eHealth and apply this in their professional work. *Teacher A1: “Although students are quite skilled in technical issues, I am often disappointed in their innovativeness.”* Students do think they are capable to work with technology and eHealth, but do not use it since they are unfamiliar with the available eHealth services and have a lack of experience of applying it. *Student M1: “I think I could apply eHealth, but in fact I know quite little about the possibilities. For this reason I will not use it just now.”*

*Attitude/ behavior towards eHealth* towards eHealth differs between individual students, according both teachers and students. Some students are highly interested in technology and choose to get more involved, whereas others do not. When discussing use of eHealth as a (future) health professional, students’ expressed it is optional, in terms of having a choice. *Student L1: “I think I will not really focus on it. I do want to know what the options are, but personally I will not devote to it for my future”. Student J1: “Once you have graduated you can make your own choice and decide whether you use it.”*

### The individual teacher

*Capabilities to use eHealth*, i.e. the knowledge about eHealth and skills on how to use eHealth, varies widely between individual teachers according to both the teachers and students. Some teachers admitted to having no overview of existing tools and eHealth interventions in their field of work or expressed to barely know how to use a projector, while others found themselves very competent as researchers in the domain of eHealth.

*Attitude and behavior towards eHealth* were rated positively towards eHealth education in general by teachers. *Teacher V1: “As a teacher I want to use eHealth in my lessons to provide ‘future proof’ education that is innovative and interactive. This makes learning more fun and challenging for students and they would be more enthusiastic about my lessons.”* However, a barrier is that teachers feel insecure about eHealth education. *Teacher K1: “I do not feel that I know enough about eHealth, but that is what I want as a teacher before I use it in my lessons.”* Students expressed that teachers have a negative attitude/behavior towards eHealth. *Student S1: “Teachers often say: find something you like, because you know better than me.” Student M2: “If a passionate teacher puts something forward, then I am more open for it. However if a teacher says ‘you are the young generation. You surely know of some app, and give it a try’, then I am not. And the latter is what I have been told so far.”*

### The social context

*Inefficient use of expertise* of eHealth within the organization was mentioned as a barrier by both teachers and students. *Student L1:* “*Teachers do not collaborate. For example get a lecturer who knows a lot about eHealth to take over the lesson. All should be benefit from it, since the less experienced teacher will catch up.” Teacher M2: “More experienced teachers who are doing research in technology or eHealth should share their knowledge with less experienced teachers. A kind of cross-fertilization”.*

*Communities of practice* were recognized as a facilitator for eHealth education according to teachers. Such communities were seen as mixed groups (teachers, students, researchers and/or the workfield) of people who share their passion and learn how to do it better by interacting regularly. *Teacher A1: “we do quite a bit of research using eHealth. It would be great if we can get an exchange between research, education and practice and in this way create an inspiring environment around eHealth.”*

*Interprofessional collaboration education* is, by both teachers and students, regarded as a facilitator for eHealth in education if students of different professions work together (e.g. technology, ICT, media, etc.). *Student N1:* “*I think it would have an enormous added value if you would work on a project on eHealth together with students with an IT background; developing an app for example.”.*

### The organizational context

*(Lack of) shared vision/rationale* in the university about what students should learn about eHealth is absent according to teachers. *Teacher D1: “As a school you have to decide to what degree you want to integrate this in your basic curricula. A choice can be to provide eHealth as a dedicated subject of choice. This can be a choice, but you will have to have an idea.”* Teachers felt that what students learn about eHealth is too much of a coincidence, depending on a students’ own interest in technology, the extent of eHealth experience during their internships/study route and the teachers they had. *“There is no clear approach for eHealth in the curricula in which connects to the professional roles (CanMeds) of the care professionals (OT/PT).”* Although there is not (yet) a clear vision about what students should learn, teachers did agree that there is a consensus within the university about the importance of eHealth education.

*Situational factors,* as mentioned by teachers, include the following barriers for eHealth education: lack of time for preparing lessons, failing technology, absence of didactic materials and relative slow curricula changes. Facilitators according to teachers would be: presence of ICT professionals within the organization, direct accessibility to materials (e.g. LivingLabs), (scheduled) time to prepare lessons, special interest group of teachers taking the lead and training for teachers to improve their competences.

### Economic and political context

*Financial aspects* are expressed by both teachers and students in 10 quotes. The lack of reimbursement by health insurance companies in the Netherlands for eHealth interventions results in the absence of incentives to use eHealth in the field of work. Teachers specifically mentioned financial aspects such as the investments in technology and eHealth, needed for eHealth education, are a financial barrier for uptake.

*The role of the government* is to provide a definition of the future health professionals in relation to eHealth to give direction for eHealth education, according to the teachers. Students expressed that it is the role of the government to manage reimbursements for eHealth interventions and to lower the workload for health professionals. According to the students, this would facilitate health professionals to apply eHealth and consequently students can experience eHealth during internships. Moreover, students want the government to improve the quality of eHealth by a national quality mark or at least a check list to determine quality of eHealth services.

*Role of profession bodies.* Students noticed during their internships eHealth is not yet imbedded in the work field. According to them, their professional organizations should facilitate uptake of eHealth in daily practice, for instance by providing education to health professionals and incorporation of eHealth in practical guidelines.

## Discussion

The aim of this study was to identify barriers and facilitators for eHealth education as perceived by teachers and students of physical therapy and functional exercise therapy. Teachers and students equally contributed to the number of facilitators and barriers for the use of eHealth in professional education identified in this focus group study. Main barriers for the *innovation* were a lack of understanding the full concept of eHealth and a lack of knowledge and skills in critically appraising eHealth. For the *individual users*, the variety in knowledge and skills of individuals was a factor influencing uptake. On the level of the *organization*, identified factors for uptake were the shared sense of importance of implementing eHealth in education, a shared vision about what students should learn about eHealth and didactic materials.. *Economical* barriers were seen in the investments in technology and eHealth. Finally, *political* factors were identified in the national government to manage future reimbursements for eHealth interventions and to improve the quality of eHealth by a quality mark.

As expected, and based on the literature, we found unanimous support for implementing eHealth in education in the curricula of two departments in the Netherlands. In line with literature, we found barriers and facilitators on all levels of implementation as described by Grol and Wensing (2014) [18]. The barriers to the uptake of the eHealth *innovation* found in our study are in line with previous research: limited skills and knowledge about the eHealth intervention in both teachers and students [5, 8, 11, 12, 14], limited confidence in working with technology in health practice [6, 8, 12] and critically appraising and applying technique [5]. Lam (2016) mentioned: ‘while students demonstrated the technical skills that would potentially enable them to engage in eHealth, they displayed a lack of understanding how these skills could be applied to professional health contexts’ [8]. On the level of the *individual user*, there is a marked diversity in knowledge and skills in both teachers and students, which is in line with previous studies [5, 8], and might hinder uptake of eHealth in the curricula.

On the level of *organization*, barriers and facilitators identified in our study add to the growing consensus that the uptake of eHealth needs a multi-facetted approach and not just ‘writing a new module’. In literature, static curricula with narrow focus on technology were reported as important barriers [7], and a clear need for a shared vision/rationale about what students should learn about eHealth and need didactic materials which is also reflected in literature [10]. *Political and economic* factors, i.e. the influential role of government, policies and professional bodies on uptake of eHealth in education, found in this study, were also reported elsewhere: this was phrased by Hilberts and Gray (2014) as the need for ‘an education infrastructure in large-scale eHealth strategies’ [14].

Strength of this study is that research in this field amongst physical therapists is relatively scare. Another strength is the structured use of the model of Grol and Wensing to gain a more in-depth knowledge of barriers and facilitators for the uptake of eHealth in education and to enable comparison between the perspectives of teachers and students. EHealth is a relatively new and emerging field and, as a consequence, so is the implementation in education. There is surprisingly little evidence for the effectiveness of education focusing on the use eHealth [14]. Last but not least, a strength is that both teachers’ and students’ perspectives were included, whilst most studies focused on single groups of teachers, students or professionals. Limitations of this study are the professional relationship of the first author with some of the teachers and students. For that reason it was made very clear to the participants that their statements were confidential and not affected their position as a teacher/student. However, some bias in their responses cannot be ruled out entirely. Moreover, further focus groups might add to the data-saturation to some degree.

For uptake of eHealth in the curricula of physical and functional exercise therapy it is eminent to recognize the multi-level character of it. This study highlighted the need for a vision on eHealth at a faculty level. Besides a generally limited understanding of the width of eHealth and the expected impact of eHealth in clinical practice, this study showed that both the lack of skill in critically appraising the quality and usefulness of eHealth and the diversity in background knowledge and skills in technology need to be a point of engagement in future uptake plans. Moreover, the ‘higher order’ influences on both education and professional practice need to be addressed in eHealth education, i.e. the role of government, policies and professional bodies. In addition, to further enhance uptake it is strongly advised to take a structured approach by addressing the levels of uptake [18]. Using ‘a clear rationale for teaching clinical informatics and a detailed list of desired competencies are an important start’ [10], and keeping in mind that ‘there is surprisingly little evidence about what works and doesn’t work with regard to the eHealth education’ [14]. Finally, more tools should be provided by the organizations itself, such as didactic materials and eHealth facilities.

This study provides insights into the many factors which influence the successful uptake of eHealth in the curricula of functional exercise and physical therapy education. This is highly important given the fact that the application of eHealth is irreversible and health professionals do not seem to be fully equipped to work with eHealth. Uptake of eHealth needs a systematic multi-facetted approach considering factors on the level of the innovation, individual users, organization and political and economic levels. Important starting points for developing uptake strategies, for both teachers and students, are a limited knowledge of eHealth, a large diversity in eHealth skills, a lack of skills in critically appraising eHealth and to development of a clear rationale for teaching eHealth. A recommendation for further research is to re-examine the study in other health professions for a good comparison of perceived barriers and facilitators for eHealth education. Moreover, future research should provide evidence for what works and doesn’t work with regard to the eHealth education.

## Conclusion

The successful uptake of eHealth in the curriculum of functional exercise/physical therapists needs a systematic multi-facetted approach, considering the barriers and facilitators for uptake identified from the perspective of teachers and students. Moreover, a relatively large amount of the identified barriers and facilitators were overlapping between teachers and students (e.g. unclear concept of eHealth, lack of quality/evidence for eHealth, negative/positive attitude of students/teachers towards eHealth), so that starting points for developing effective implementation strategies can potentially be found in those overlapping barriers and facilitators.

## Additional file

Additional file 1: Results on codes, (sub)themes and levels of perceived barriers (B) and facilitators (F) for eHealth education according to teachers and students. (DOCX 18 kb)

## Data Availability

The datasets used and/or analyzed during the current study are available from the corresponding author on reasonable request.
