# Supplementary material for: Teachers’ and students’ perceptions on barriers and facilitators for eHealth education in the curriculum of functional exercise and physical therapy: a focus groups study
Source: BMC Med Educ. 2019 Sep 6;19:343. doi: 10.1186/s12909-019-1778-5 (PMC6731570; doi:10.1186/s12909-019-1778-5)
Supplement: Supplementary file 1 — Results on codes, (sub)themes and levels of perceived barriers (B) and facilitators (F) for eHealth education according to teachers and students. (DOCX 18 kb) [file 12909_2019_1778_MOESM1_ESM.docx]

**Additional file 1. Results on codes, (sub)themes and levels of perceived barriers (B) and facilitators (F) for eHealth education according to teachers and students.**

|  |  | | | **Teachers** | | **Students** | |
| --- | --- | --- | --- | --- | --- | --- | --- |
| **Codes**  (n= 109) | **Levels** | **Themes** | **Subthemes** | **B** | **F** | **B** | **F** |
| 26 | **Innovation** | *Unclear concept of eHealth (education)* | Not aware of the definition | X |  | X |  |
|  |  |  | Not aware of the possibilities of eHealth (available tools and interventions) | X |  | X |  |
|  |  |  | No attention for motivation when, how and why to use eHealth in the treatment process of a patient | X |  | X |  |
|  |  |  | Not able to support patients with use of eHealth | X |  |  |  |
|  |  | *Lack of a quality mark and evidence for eHealth services* | Lack of a quality mark for eHealth services (i.e. applicability, usability, content) | X |  | X |  |
|  |  |  | Safety and privacy of a patient | X |  |  |  |
|  |  |  | No quality mark for eHealth services (reliability and safety) | X |  | X |  |
|  |  |  | Rapid developments in available eHealth | X |  | X |  |
|  |  |  | A lack of evidence for eHealth interventions in patient groups | X |  | X |  |
|  |  |  | No integration of effective eHealth services in practical guidelines | X |  | X |  |
| 17 | **Individual student** | *Capabilities of student on how to use eHealth* | (Lack of) experience with technology and/or eHealth services | X | X | X | X |
|  |  |  | Lack of knowledge how eHealth can be beneficial for treatment of patients |  |  | X |  |
|  |  |  | A lack of knowledge of eHealth in general (evidence, what is available and how it can be used) |  |  | X |  |
|  |  |  | Although experience with technology, not able to innovate as a professional | X |  |  |  |
|  |  | *Attitude/behavior of students towards eHealth* | Interest and affinity with technology and eHealth |  | X | X | X |
|  |  |  | Want to be a future proof health professionals |  |  |  | X |
|  |  |  | Want to be prepared for graduation |  |  |  | X |
| 22 | **Individual teacher** | *Capabilities of teachers on how to use eHealth* | A lack of knowledge of eHealth in general (evidence, what is available and how it can be used) | X | X | X |  |
|  |  |  | A lack of skills on how to use technology and eHealth | X | X | X |  |
|  |  |  | Lack of capabilities on how to use eHealth in education | X |  |  |  |
|  |  | *Attitude/behavior of teachers towards eHealth (education)* | (Lack of) affinity with technology and eHealth |  | X | X |  |
|  |  |  | Providing interactive education that is fun and challenging for students by applying eHealth |  | X |  |  |
|  |  |  | Teaching for the future by applying eHealth |  | X |  |  |
|  |  |  | Feeling insecure about eHealth education | X |  |  |  |
|  |  |  | Students are more skillful then teachers when it comes to technology and eHealth | X |  | X |  |
| 14 | **Social context** | *Inefficient use/not sharing of expertise* | No efficient use of knowledge between teachers and students | X |  | X |  |
|  |  | *Communities of practice* | Share experiences and knowledge about eHealth in communities of students, teachers, researchers and health care professionals |  | X |  |  |
|  |  |  | Intensive collaboration between research, work practice and education within communities of practice |  | X |  |  |
|  |  | *Interprofessional collaboration and education* | (Lack of) working together with other disciplines (e.g. technology, ICT, media, etc.) | X | X | X | X |
| 20 | **Organizational context** | *(Lack of) a shared vision/rationale within the organization* | No competences on the level of CanMeds | X |  |  |  |
|  |  |  | No alignment of eHealth education throughout the entire curriculum | X |  |  |  |
|  |  |  | A shared sense of importance for eHealth education. |  | X |  |  |
|  |  | *Situational factors (e.g. lack of time, slow curriculum changes)* | Learning about eHealth is too much of a choice and/or coincidence (depends on a students’ own interest, study route/internships and teachers) | X |  |  |  |
|  |  |  | Lack of time for preparation of lessons | X |  |  |  |
|  |  |  | Relative slow curriculum changes | X |  |  |  |
|  |  |  | Absence of didactic materials for eHealth education | X |  |  |  |
|  |  |  | Failing technology and a lack of available technology and eHealth to use in lessons | X |  |  |  |
|  |  |  | Training for teachers on how to use eHealth and for eHealth education |  | X |  |  |
|  |  |  | Presence of ICT professionals within the organization ICT helpdesk |  | X |  |  |
|  |  |  | Training for teachers to improve their competences |  | X |  |  |
|  |  |  | (Scheduled) time to prepare lessons |  | X |  |  |
|  |  |  | Special interest groups of teachers taking the lead |  | X |  |  |
|  |  |  | Direct accessibility to materials (e.g. LivingLabs, a lab with technology and eHealth) |  | X |  |  |
| 10 | **Political and economic factors** | *Financial aspects* | Expensive to purchase technology and eHealth |  | X |  |  |
|  |  |  | Time investments to learn how to use eHealth and develop new lessons |  | X |  |  |
|  |  |  | No reimbursement for use of eHealth in health care | X |  | X |  |
|  |  | *Role of government* | Provide a definition of the future health professionals in relation to eHealth to give direction for eHealth education |  | X |  |  |
|  |  |  | Lower the workload for health professionals |  |  |  | X |
|  |  |  | Manage reimbursements for eHealth interventions |  |  |  | X |
|  |  |  | Improve the quality of eHealth by a national quality mark or at least a check list to determine quality of eHealth |  |  |  | X |
|  |  | *Role of profession bodies* | Providing education for allied health professionals |  |  |  | X |
|  |  |  | Incorporation of eHealth in practical guidelines. |  |  |  | X |
